# Shotgun Immunoproteomic Approach for the Discovery of Linear B-Cell Epitopes in Biothreat Agents *Francisella tularensis* and *Burkholderia pseudomallei*

**DOI:** 10.3389/fimmu.2021.716676

**Published:** 2021-09-29

**Authors:** Patrik D’haeseleer, Nicole M. Collette, Victoria Lao, Brent W. Segelke, Steven S. Branda, Magdalena Franco

**Affiliations:** ^1^ Biosciences and Biotechnology Division, Lawrence Livermore National Laboratory, Livermore, CA, United States; ^2^ Molecular and Microbiology Department, Sandia National Laboratories, Livermore, CA, United States

**Keywords:** *Francisella*, *Burkholderia*, immunoproteome, B-cell epitope, antigen, peptide vaccine

## Abstract

Peptide-based subunit vaccines are coming to the forefront of current vaccine approaches, with safety and cost-effective production among their top advantages. Peptide vaccine formulations consist of multiple synthetic linear epitopes that together trigger desired immune responses that can result in robust immune memory. The advantages of linear compared to conformational epitopes are their simple structure, ease of synthesis, and ability to stimulate immune responses by means that do not require complex 3D conformation. Prediction of linear epitopes through use of computational tools is fast and cost-effective, but typically of low accuracy, necessitating extensive experimentation to verify results. On the other hand, identification of linear epitopes through experimental screening has been an inefficient process that requires thorough characterization of previously identified full-length protein antigens, or laborious techniques involving genetic manipulation of organisms. In this study, we apply a newly developed generalizable screening method that enables efficient identification of B-cell epitopes in the proteomes of pathogenic bacteria. As a test case, we used this method to identify epitopes in the proteome of *Francisella tularensis* (Ft), a Select Agent with a well-characterized immunoproteome. Our screen identified many peptides that map to known antigens, including verified and predicted outer membrane proteins and extracellular proteins, validating the utility of this approach. We then used the method to identify seroreactive peptides in the less characterized immunoproteome of Select Agent *Burkholderia pseudomallei* (Bp). This screen revealed known Bp antigens as well as proteins that have not been previously identified as antigens. Although B-cell epitope prediction tools Bepipred 2.0 and iBCE-EL classified many of our seroreactive peptides as epitopes, they did not score them significantly higher than the non-reactive tryptic peptides in our study, nor did they assign higher scores to seroreactive peptides from known Ft or Bp antigens, highlighting the need for experimental data instead of relying on computational epitope predictions alone. The present workflow is easily adaptable to detecting peptide targets relevant to the immune systems of other mammalian species, including humans (depending upon the availability of convalescent sera from patients), and could aid in accelerating the discovery of B-cell epitopes and development of vaccines to counter emerging biological threats.

## Introduction

Development of an effective vaccine against a biothreat agent or emerging pathogen is a costly and cumbersome process that can take years to decades to complete. The identification of antigens that stimulate protective immunity against a pathogen can represent a significant bottleneck in the vaccine development process, especially for bacterial or fungal pathogens, eukaryotic parasites, or even large DNA viruses, which can contain hundreds to thousands of potential antigens. Our study addressed the need to accelerate this process by testing the feasibility of a screening platform for efficient identification of immunoreactive peptides that could be utilized as candidates for development of peptide-based vaccines.

Peptide-based vaccines represent a potential solution to provide protection against biothreat and emerging pathogens to which current vaccine development strategies have failed. Peptide vaccine formulations consist of multiple synthetic linear epitopes that together trigger immune responses resulting in robust immune memory. This multi-epitope, multi-target approach has the potential to be broadly protective across divergent strains (e.g., the first universal influenza vaccine to enter phase III clinical trials was a peptide vaccine), and could be effective for pathogens with complex life cycles (e.g., several malaria peptide vaccines are currently in clinical trials) (1–3). Although it has been reported that conformational (discontinuous) epitopes make up the majority of B-cell epitopes (4), linear epitopes possess several advantages for vaccine design over conformational epitopes. Due to their short sequence and lack of complex secondary and tertiary structure, short antigenic peptides can be easily synthesized, and multiplexed into vaccine formulations, for high-throughput assessment of efficacy. Consequently, peptide-based vaccines are potentially powerful medical countermeasures that would seem amenable to rapid development in responding to infectious disease outbreaks.

Current strategies for epitope identification depend upon detection of epitopes within an individual full-length protein, a low-throughput approach that requires prior knowledge of the antigenic protein, its sequence, and its conformational structure. Technologies to screen for epitopes at the whole proteome level have been developed (e.g., proteomic microarrays, phage and yeast display); however, these technologies require extensive use of synthetic biology and other time-consuming methodologies (e.g., library construction, peptide/protein array preparation, heterologous protein expression) (3, 5–11). Another major disadvantage of display technologies and use of non-native expression systems is that these methods do not reliably replicate the native properties of the antigenic proteins, including their post-translational modifications, which can lead to inaccurate identification of epitopes.

In this study, proteome-wide screening for linear B-cell epitopes was achieved using total protein extracts isolated from the pathogen of interest, affinity purified using antibodies from convalescent sera from infected animals. This strategy holds several advantages over the currently available methods for epitope discovery: It does not require prior knowledge of antigenicity or antigen structure, and obviates need for complex and laborious experimental techniques such as preparation of display libraries and heterologous protein expression. As with other methods for epitope discovery from serum, it may be less well suited for pathogens for which natural infection does not confer immunity, such as HIV, malaria and TB, although even in those cases protective antibodies may be found in some subsets of patients or animal models (12–14).

Our approach was designed to enable identification of the protein antigen and, importantly, the antigenic regions within the identified antigen, such that these short linear peptides can be immediately synthesized and tested for efficacy in vaccine formulations. Note that several strategies have been previously developed for the identification of T-cell peptide epitopes (15, 16), including techniques similar to that presented here involving purification of MHC-bound peptides and their subsequent identification *via* LC/MS/MS (17).

In this study, we focused on two intracellular bacterial pathogens, *Francisella tularensis* (Ft) and *Burkholderia pseudomallei* (Bp), organisms which pose a high risk for misuse as bioweapons and therefore are considered Tier 1 Select Agents by the US Centers for Disease Control and Prevention. The mortality rates of both pathogens are high, and there is currently no licensed vaccine available for either agent (18–20). Humoral immunity plays an important role in developing immune protection to both of these intracellular pathogens, making them good model organisms for the purposes of this study (21–26). In addition, the immunoproteome of Ft has been thoroughly characterized (19, 27, 28), such that the previously published data could be compared to the datasets generated in our study. We leveraged a merged dataset of 164 previously identified antigens, corresponding to ~10% of Ft proteome. The Bp immunoproteome is not as well characterized compared to that of Ft: our reference dataset contained only 61 previously identified seroreactive proteins, corresponding to ~1% of the Bp proteome (29, 30). Consequently, analysis of the dataset resulting from the Bp screen has revealed many proteins that have not been previously categorized as antigens.

## Materials and Methods

### Bacterial Strains and Culture Conditions

*Francisella tularensis* SCHU S4ΔclpB (“Ft-ΔclpB”) was a generous gift from Dr. Wayne Conlan (National Research Council Canada). Stock cultures were prepared by growing Ft-ΔclpB on Chocolate II Agar plates supplemented with hemoglobin and isovitalex (BD 221169) for 48 hours at 37°C. Bacteria were harvested by scraping confluent lawns into Mueller Hinton (MH) broth containing 20% (w/v) sucrose, and stored at -80°C at a concentration 108 - 109 CFU/mL. *Burkholderia pseudomallei* mutant ΔpurM (“Bp82”) was obtained from BEI resources (NR-51280). Frozen stocks were prepared by growing the bacteria to log phase in Luria-Bertani (LB) broth, adding glycerol to achieve 20% (w/v) with the bacteria at a final concentration of 10^8^ - 10^9^ CFU/mL, and storing aliquots at -80°C. For immunizations, the Ft-ΔclpB and Bp82 bacterial stocks were thawed and diluted in sterile phosphate-buffered saline (PBS) to the specified concentrations used for dosing. For protein extraction purposes, Ft-ΔclpB and Bp82 were propagated to log phase in MH and LB broth, respectively. Both bacterial strains used in this study are classified as Risk Group 2 organisms. All biological materials were handled under standard institutional biosafety and biosecurity procedures, as outlined in an approved Institutional Biosafety Committee (IBC) protocol.

### Protein Extraction and Peptide Preparation

Ft-ΔclpB and Bp82 were grown to log phase in 300 mL of MH broth or LB broth, respectively, at 37°C with shaking (250 rpm). The bacteria were harvested by centrifugation at 3200 x g for 10 min at 4°C, washed once with 10 mL of PBS, and the pellet flash frozen using dry ice. The bacteria in the pellet were lysed by subjecting them to two freeze-thaw cycles (alternating between room temperature and dry ice). For protein extraction, the lysate was mixed with Bper Complete Bacterial Protein Extraction Reagent (Thermo Fisher Scientific cat# 89822), and the mixture incubated at room temperature for 15 min with rotational shaking. The mixture was then subjected to two rounds of sonication (1 sec pulses, timed output 10 sec, at 50% power) using a Heat Systems Ultrasonics sonicator (model W-385), and centrifuged at 16,000 x g for 10 min. Proteins were precipitated with acetone and washed twice with ethanol. Air-dried protein pellets were solubilized using 8M urea and Protease Max surfactant (Promega cat# V2071), then digested with trypsin (Promega cat# V5111) using the in-solution digestion protocol provided by the manufacturer (Promega cat# TB373). Completion of the trypsinization reaction was confirmed by gel electrophoresis. The trypsin-digested proteins were filtered using 10K MWCO concentrators (Pierce) at 10,000 x g for 20 min at 20°C, and the filtrates (purified peptides) stored at -20°C. These purified peptides preparations were used as inputs in subsequent experiments.

### Mice and Immunizations

Mouse immunization studies were carried out in strict accordance with the recommendations in the Guide for the Care and Use of Laboratory Animals and the National Institutes of Health. Standard institutional safety and biosecurity procedures were followed for *in vivo* experiments. Appropriate efforts were made to minimize suffering of animals. All animals were housed in ABSL2 conditions in an AAALAC-accredited facility, and the protocol (Protocol 270, renumbered 284) was approved by the LLNL Institutional Animal Care and Use Committee (IACUC). For immunization, 6 week-old female specific-pathogen-free BALB/c-Elite and C57BL/6J-Elite mice (Charles River) were injected subcutaneously with 10^6 CFU Ft-ΔclpB (BALB/c and C57BL/6J), or intradermally with 10^7 CFU Bp82 (BALB/c), and boosted at 2 weeks. No adjuvants were used. Matched PBS-dosed controls were included for each injection route. Course of infection was monitored by performing daily health scoring and weight measurements. Mice that developed infection wounds (Ft only) were topically treated with Dakin’s solution to encourage wound healing, and allowed to remain on test so long as they did not meet humane endpoint criteria (any mice with ~20% body weight loss or overt signs of morbidity were humanely euthanized). Sera from euthanized mice were excluded from analysis due to lack of immunity to the pathogen. Convalescent sera were harvested from resilient mice at 4 weeks post-infection, *via* cardiac puncture terminal bleeding under inhaled isoflurane anesthesia followed by blood fractionation [centrifugation at 3800 x g for 15 min in microtainer serum separator tubes (BD)]. Sera were stored at -80°C.

### SDS-PAGE and Western Analysis

Western analysis was performed to confirm seropositivity of infected mice. Bacterial lysates were prepared using Bper Complete Bacterial Protein Extraction Reagent (Thermo Fisher Scientific cat# 89822), combined with Laemmli loading buffer (BioRad), and boiled at 95°C for 5 min. Samples were loaded onto 4-15% acrylamide gels (Mini-Protean TGX, BioRad) and separated by electrophoresis at 120 V for 1 hr. The proteins were transferred from the gels to nitrocellulose membranes (BioRad). Membranes were blocked with Tris-buffered saline plus 0.05% Tween 20 (TBS-T) plus 5% nonfat dry milk, at room temperature for 1 hr or at 4°C for 16 hrs. The membranes were hybridized with mouse sera at 1:500 dilution in TBS-T plus 5% milk, at room temperature for 2 hrs; washed three times with TBS-T; and then incubated with goat anti-mouse antibodies conjugated to HRP (Pierce cat# 1858413), at 1:5000 dilution in TBS-T plus 5% milk, at room temperature for 1 hr. After three TBS-T washes, the membranes were developed using SuperSignal™ West Pico PLUS Chemiluminescent Substrate (Thermo Fisher Scientific).

### Enzyme-Linked Immunosorbent Assay

ELISA was performed to assess the level of seropositivity of infected mice. Wells were coated with bacterial lysates and incubated at 4°C for 16 hrs. After three washes with PBS plus 0.1% Tween-20 (PBS-T), sera from infected mice diluted to 1:100 with PBS were added to the wells and incubated at room temperature for 1 hr. Following four PBS-T washes, the wells were incubated for 1 hr with Recombinant Protein A/G peroxidase (Pierce cat# 32490) diluted at 1:5000 with PBS. After four PBS-T washes, 1-Step ABTS Substrate Solution (Pierce cat# 37615) was added, and after 15 min incubation any colorimetric changes in the wells were detected using a microplate reader (Tecan M200 Pro).

### Affinity Purification of Immunoreactive Peptides

Magnetic beads coated with protein G (Invitrogen cat# 10007D) were used to capture antibodies from pools of sera obtained from either infected (experiment) mice or mock-infected (control) mice, following the manufacturer’s protocol (MAN0017348). Each pool was comprised of sera recovered from 3-5 mice, with equal volumes used for each experiment-control pair. The antibody-coated beads were then incubated with peptide preparations (inputs) at room temperature for 45 min. Antibody-coated beads from each experiment-control pair were incubated with the same input peptides; in total, 6 input peptide preparations were used with the 8 Ft experiment-control pairs, and 5 with the 9 Bp experiment-control pairs. Following three PBS washes, immunoreactive peptides were eluted from the beads using citrate buffer (pH 3). Input, unbound, and eluted (output) peptides were flash frozen with dry ice and stored at -20°C.

### Mass Spectrometry

The input, unbound, and eluted (output) peptides recovered from the antibody-coated beads (see preceding section) were desalted using an Empore SD solid phase extraction plate; lyophilized; reconstituted in 0.1% TFA; and analyzed *via* LC-MS/MS by MS Bioworks (Ann Arbor, Michigan), using a Waters M-Class UPLC system interfaced to a ThermoFisher Fusion Lumos mass spectrometer. Peptides were loaded on a trapping column and eluted over a 75 μm analytical column at 350 nL/min. Both columns were packed with Luna C18 resin (Phenomenex). A 2 hr gradient was employed. The mass spectrometer was operated in a data dependent HCD mode, with MS and MS/MS performed in the Orbitrap at 60,000 FWHM resolution and 15,000 FWHM resolution, respectively. The instrument was run with a 3 sec cycle for MS and MS/MS.

### MS Data Processing

Data were analyzed using Mascot (Matrix Science) with the following parameters: Enzyme: Trypsin/P; Database: UniProt *F. tularensis* SCHU S4 or UniProt *B. pseudomallei* strain 1026b (forward and reverse appended with common contaminants and mouse IgG sequences); Fixed modification: Carbamidomethyl (C); Variable modifications: Oxidation (M), Acetyl (N-term), Pyro-Glu (N-term Q), Deamidation (N/Q); Mass values: Monoisotopic; Peptide Mass Tolerance: 10 ppm; Fragment Mass Tolerance: 0.02 Da; Max Missed Cleavages: 2; Mascot DAT files were parsed into Scaffold Proteome Software for validation, filtering and to create a non-redundant list per sample. Data were filtered using 1% protein and peptide FDR and requiring at least one unique peptide per protein.

### Bioinformatic Analysis

Each experiment typically consisted of three sets of data: “Input” (total bacterial peptides without affinity purification), “Control” (peptides purified from beads coated with antibodies from uninfected mice), and “Experiment” (peptides purified from beads coated with antibodies from infected mice).

LC-MS/MS data were analyzed at the peptide level, rather than rolling up peptide scores into a protein abundance metric as would be done in standard proteomics. We used the Total Ion Current (TIC, total area under the MS2 curve) as a metric for the abundance of the peptide in each sample. Input datasets were first normalized against each other based on median ratios for the peptides occurring in every Input dataset. The sparser Control and Experiment datasets were then normalized against their respective Input dataset based on median ratios as well. Since each animal can be expected to raise a different set of antibodies, we counted how often specific output peptides occurred more abundantly in the Experiment *vs* Control, rather than focusing on the average log fold change in abundance. For each peptide and each Experiment sample, we assigned an enrichment score of +1, 0, or -1 depending on whether the normalized peptide abundance was greater than, equal to, or lower in the Experiment than in the corresponding Control sample, creating a score matrix of peptides × Experiments. The total enrichment score for each peptide is then the sum of its enrichment scores across each Experiment. Statistical significance was evaluated by generating a number of randomized score matrices, where each peptide was randomly assigned a +1, 0, or -1 score for each Experiment, with the same probabilities as in the real matrix, and calculating how frequently peptides reach an specific total enrichment score. This gives us a background level of how many high-scoring peptides we would expect to see even if there was no correlation in peptide abundance across the different experiments, which can then be used to calculate the significance level of observing a given number of high scoring peptides in the real data, using a simple binomial test comparing expected *vs* observed number of peptides exceeding a given score.

Amino Acid Conservation Scores were calculated using the ConSurf web server (31) with default parameter values, using near full-length protein structure homology models from SWISS-MODEL or crystal structures from PDB where available. These scores are normalized position-specific evolutionary rates, with negative scores indicating the most conserved amino acids. The Average Amino Acid Conservation Score (AAACS), proposed by Ren et al. as a useful tool to identify conserved epitopes that may be targeted by broadly neutralizing antibodies, is the average of the conservation score for the residues in an epitope, with negative scores indicating more highly conserved regions (32).

In addition to AAACS, we also scored peptides based on how many complete sequenced genomes of pathogenic *B. pseudomallei* and *F. tularensis* they occurred in, similar to the conservation analysis in EpitoCore (33). We downloaded proteomes for all 110 *B. pseudomallei* strains with complete genome sequences available through NCBI. For *F. tularensis*, 36 strains with complete genomes were available through NCBI, but several of these corresponded to the less-pathogenic *novicida, holartica* and *mediasiatica* subspecies, so we decided to focus exclusively on the 17 available *F. tularensis* subsp. *tularensis* complete genomes. We identified homologs with ≥90% sequence identity to the proteins containing our top scoring peptides in **Tables 1**, **2**, and then scored each peptide based on how often they had a 100% identical hit in each homolog.

**Table 1:** List of top scoring immunoreactive peptides identified for *Francisella tularensis*.

Protein name	Accession	Peptide	Scores							
Aminotransferase AspC1	Q5NGG1	LPIDDAEK^2^								
**Glutamate dehydrogenase Gdh**	Q5NHR7^a^	FHPSVYSGIIK								
Pyruvate dehydrogenase acetyltransferase AceF	Q5NEX3^a^	VSQGSLILK^2^								
**60 kDa chaperonin GroL**	Q5NEE1^a^	DRVDDALHATR^2^								
**Chaperone protein DnaK**	Q5NFG7^a^	NTADNLIHSSR								
**Chaperone protein DnaK**	Q5NFG7^a^	SSSGLSEEDIEK								
**60 kDa chaperonin GroL**	Q5NEE1^a^	DNTTIIDGAGEK								
**60 kDa chaperonin GroL**	Q5NEE1^a^	EGVITVEEGK								
**Catalase-peroxidase KatG**	Q5NGV7^a^	AVAQVYAENGNEQK								
Malate dehydrogenase Mdh	Q5NHC8^a^	FSGVPDNK^1^								
**Outer membrane protein 26 Omp26**	Q5NES2°	EIPADQLGTIK								
Succinate dehydrogenase flavoprotein SdhA	Q5NIJ3^a,i^	ITILATGGAGR								
**ATP synthase subunit alpha AtpA**	Q5NIK5^a^	GEVATDLTSPIEK								
**Elongation factor Ts Tsf**	Q5NHX9^a^	ESGKPAEIIEK								
**Elongation factor Ts Tsf**	Q5NHX9^a^	TVEAETLGAYIHGSK								
**Chaperone protein DnaK**	Q5NFG7^a^	IAGLEVK^1^								
Cell division protein FtsZ	Q5NI93^a^	KETEVVTGASNAPK								
Trigger factor Tig	Q5NH48	GGVDTFENEIK								
**ATP synthase subunit alpha AtpA**	Q5NIK5^a^	SVDQALQTGIK								
**Catalase-peroxidase KatG**	Q5NGV7^a^	NDNLSPQSVDLSPLR								
Isocitrate dehydrogenase [NADP] Idh	Q5NET6^a^	VADIELETK^2^								
Fructose-1,6-bisphosphate aldolase FbaB	Q5NF78^a^	KINIDTDLR								
**Glutamate dehydrogenase Gdh**	Q5NHR7^a^	GFVHDPEGITTDEK								
**Succinate–CoA ligase [ADP-forming] beta SucC**	Q5NHF3^a^	PANFLDVGGGATK^1^								
**Chaperone protein DnaK**	Q5NFG7^a^	KVPYAVIK^2^								
Malonyl CoA-ACP transacylase	Q5NF69^a^	EPTTAVVQNFDAK								
Peroxiredoxin	Q5NHA9^a^	KVPNVTFK^2^								
**Chaperone protein DnaK**	Q5NFG7^a^	IINEPTAAALAYGVDSK								
Conserved hypothetical lipoprotein LpnA	Q5NGE4^a,o^	ATVYTTYNNNPQGSVR								
**Elongation factor Tu Tuf**	Q5NID9^a^	TTVTGVEMFR								
**Succinate–CoA ligase [ADP-forming] beta SucC**	Q5NHF3^a^	EVAESLIGK^1^								
30S ribosomal protein S1 RpsA	Q5NI98^a^	KIELWDR^2^								
**Elongation factor Tu Tuf**	Q5NID9^a^	HYAHVDCPGHADYVK^1^								
Transcription elongation factor GreA	Q5NFC6^a^	IVGEDEADIDNQK								
**60 kDa chaperonin GroL**	Q5NEE1^a^	SFGTPTITK^2^								
**Aconitate hydratase AcnA**	Q5NII1^a^	GIPLVILAGK^1^								
**Chaperone protein DnaK**	Q5NFG7^a^	AYAEQAQAAVAQGGAK								
**Chaperone protein DnaK**	Q5NFG7^a^	FHDLVTAR^2^								
**Outer membrane protein 26 Omp26**	Q5NES2	DGSVGWVK^1^								
3-oxoacyl-ACP reductase FabG	Q5NF68	VALVTGASR^1^								
**Chaperone protein DnaK**	Q5NFG7^a^	ALEDAGLSK^2^								
Enoyl-ACP reductase [NADH] FabI	Q5NGQ3^i^	TLAASGISNFK								
**Aconitate hydratase AcnA**	Q5NII1^a^	TAHTTTFEALAR								
**Elongation factor Ts Tsf**	Q5NHX9^a^	LDVGEGIEK^1^								

The columns under “scores” indicate whether the peptide was over or underrepresented in each of the 8 experimental samples compared to its control sample. Blue: experiment>control. Red: experiment<control. White: peptide undetected in both experiment and control. Dark colors indicate >2-fold difference in relative abundance. Proteins with multiple top scoring peptides are highlighted in bold. See also **Supplementary Table S1** for an extended version of this table.

^a^known antigen, ^i^inner membrane,°outer membrane.

^1^peptide sequence is only a single amino acid away from a human or mouse peptide. ^2^peptide is only two amino acids away from a human or mouse peptide.

**Table 2:** List of top scoring immunoreactive peptides identified for *Burkholderia pseudomallei*.

Protein name	Accession	Peptide	Scores								
**Aspartate–tRNA(Asp/Asn) ligase AspS**	A0A0H3HT48	TGAQDGDIIFFAADR									
Adenylosuccinate synthetase PurA	A0A0H3HJJ2	QDQIGITLANVGK									
Dihydrolipoyl dehydrogenase OdhL	A0A0H3HQK7	FPFSINGR^2^									
Ankyrin repeat-containing protein	A0A0H3HJC7	IGDAPAPNAQK									
Phosphoribosylformylglycinamidine synthase PurL	A0A0H3HPH9	GATETFVVLPR									
DNA-directed RNA polymerase subunit beta RpoB	A0A0H3HT47	STGPYSLVTQQPLGGK									
**50S ribosomal protein L6 RplF**	A0A0H3HQ22	GYRPPEPYK									
DNA-directed RNA polymerase subunit beta RpoC	A0A0H3HP07	ISLYATTVGR									
**Enolase Eno**	A0A0H3HLA6	GIANSILIK^2^									
Uncharacterized protein	A0A0H3HWA2	IDCLTNAYTAR									
DNA gyrase subunit A GyrA	A0A0H3HKL0	INVVLPVR^2^									
Aspartate-semialdehyde dehydrogenase Asd	A0A0H3HW74	VTGTLSVPVGR									
Malic enzyme	A0A0H3HP28	AALLSNSNFGSAPSASSR									
50S ribosomal protein L10 RplJ	A0A0H3HUR4	AQTVVLAEYR									
**50S ribosomal protein L6 RplF**	A0A0H3HQ22	AIIANAVHGVTK									
Glutamine synthetase GlnA	A0A0H3HL61	ALNAITNPTTNSYK									
Nucleoside diphosphate kinase Ndk	A0A0H3HJK0^e^	NVIGQIYSR^2^									
Antioxidant protein LsfA	A0A0H3HGZ9	LIITYPASTGR									
UDP-glucose 4-epimerase	A0A0H3HFV2	GYSVLEVVR									
**Enolase Eno**	A0A0H3HLA6	SAIVDIIGR^2^									
Acetyl-CoA acetyltransferase	A0A0H3HTT4	LPLSVGCTTINK									
KHG/KDPG aldolase Eda	A0A0H3HGE0	FGVSPGLTR^2^									
10 kDa chaperonin GroES	A0A0H3HH83^a^	TASGIVIPDAAAEKPDQGEVLAIGPGKR									
Saccharopine dehydrogenase	A0A0H3HIF5	HGQLVQDVFTR									
Citrate synthase GltA	A0A0H3HYU5	YSIGQPFVYPR									
**Aspartate–tRNA(Asp/Asn) ligase AspS**	A0A0H3HT48	YVAAHHPFTSPK									
Gamma-aminobutyraldehyde dehydrogenase	A0A0H3HQU5	SVLAAAAGNLK^2^									
Peptide chain release factor 2 PrfB	A0A0H3HL96	SYVLDQSR^2^									
Polyketide non-ribosomal peptide synthase	A0A0H3HWL5^i^	AWFIPLSAR^2^									
Transcription termination/antitermination NusG	A0A0H3HPU8	VTGFVGGAR^2^									
Beta sliding clamp DnaN	A0A0H3HFM1	FTFGQVELVSK									
Malate synthase AceB	A0A0H3HIT5	IATLIVRPR^2^									
PTS system, EIIA component	A0A0H3HRL4	ISGHHLEVTPAIR									
Phosphoenolpyruvate synthase PpsA	A0A0H3HJ13	IFILQARPETVK									
Thiol:disulfide interchange protein DsbA	A0A0H3HTS6^p^	NYNIDGVPTIVVQGK									
RND family efflux transporter MFP subunit BpeA	A0A0H3HQZ3^i^	AQANLATQNALVAR									
Inosine-5’-monophosphate dehydrogenase GuaB	A0A0H3HJ23	LVGIVTNR^1^									
Periplasmic maltose-binding protein MalE	A0A0H3HG39^p^	VNWLYINK									
Putative extracellular ligand binding protein	A0A0H3HWC6^p^	VVATDAQQGPALADYAK									
Acid phosphatase AcpA	A0A0H3HV11^e^	NIVVIYAENR									
NADH-quinone oxidoreductase subunit F NuoF	A0A0H3HPW5	EGTGWLYR^2^									
Type VI secretion system Hcp-1	A0A0H3HE88^e^	IGGNQGGNTQGAWSLTK									
50S ribosomal protein L23 RplW	A0A0H3HT35	AAVELLFK^2^									
**50S ribosomal protein L6 RplF**	A0A0H3HQ22	LTLVGVGYR									
50S ribosomal protein L17 RplQ	A0A0H3HPQ2	LFDVLGPR^2^									
Aconitate hydratase	A0A0H3HVV9	IVLESVLR^1^									

The columns under “scores” indicate whether the peptide was over or underrepresented in each of the 9 experimental samples compared to its control sample. Blue: experiment>control. Red: experiment<control. White: peptide undetected in both experiment and control. Dark colors indicate >2-fold difference in relative abundance. Proteins with multiple top scoring peptides are highlighted in bold. See also **Supplementary Table S2** for an extended version of this table.

^a^known antigen, ^i^inner membrane, ^p^periplasmic, ^e^extracellular.

^1^peptide sequence is only a single amino acid away from a human or mouse peptide. ^2^peptide is only two amino acids away from a human or mouse peptide.

We used two state-of-the-art computational B-cell epitope prediction tools to evaluate all of the peptides in our proteomic data that match the proteins in **Tables 1**, **2**. Peptides were submitted to the iBCE-EL web server for scoring (34). iBCE-EL is an ensemble-based method based on extremely randomized tree and gradient boosting classifiers, trained on 5,550 experimentally validated B-cell epitopes and 6,893 non-epitopes from the Immune Epitope Database, to identify linear B-cell epitopes. In addition, proteins were submitted to the Bepipred Linear Epitope Prediction 2.0 tool on the IEDB website (35), and peptides were then scored based on their average predicted residue score. Bepipred 2.0 is a random forest classifier trained on 160 non-redundant antigen-antibody crystal structures, to predict the probability that a given antigen residue is part of an epitope.

## Results

### Overview of Immunoproteome Screen

**Figure 1:**
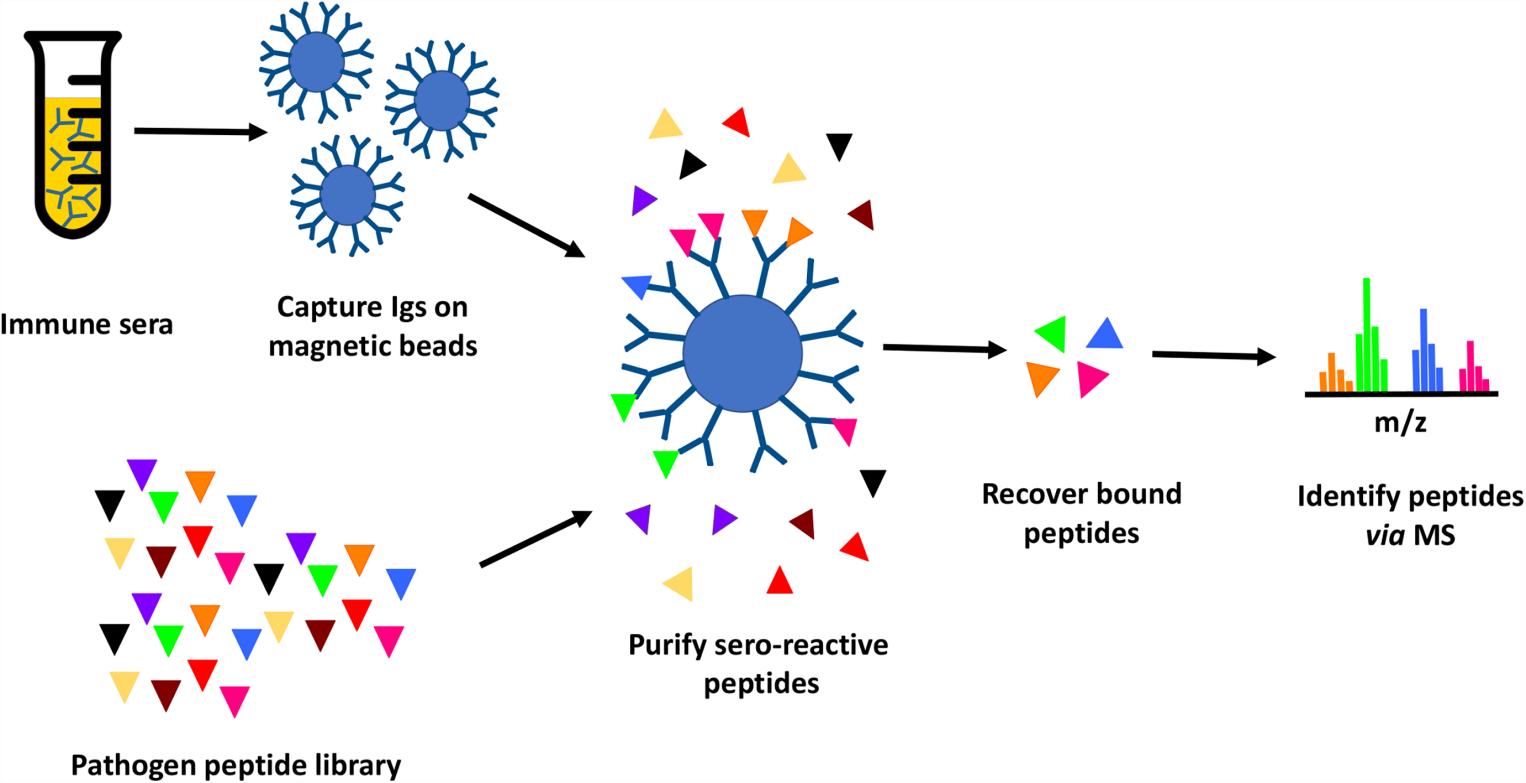
Immunoproteome screening workflow. Schematic overview of high throughput approach for identification of seroreactive peptides in the proteomes of pathogens.

In this study, we tested the feasibility of proteome-wide screening for linear B-cell epitopes using peptide extracts from target bacteria and sera from infected animals. The method requires: (1) isolation of peptides from lysates generated from the target bacteria; (2) challenge of the host (in this case, mouse) with the target bacteria, followed by collection of convalescent serum; (3) mixing of the bacterial peptides and convalescent serum, to allow peptide antigens to bind to their cognate antibodies in the serum; and (4) recovery of bound peptides for identification through mass spectrometry (**Figure 1**). We applied this method to two bacterial Select Agent pathogens: *Francisella tularensis* and *Burkholderia pseudomallei.* Infection with attenuated strains of these pathogens [*F. tularensis* SCHU S4ΔclpB and *B. pseudomallei* ΔpurM (strain Bp82)] has been shown to stimulate development of protective immunity against their corresponding fully-virulent parental strains (*F. tularensis* SCHU S4 and *B. pseudomallei* K96245, respectively) (36, 37), suggesting that convalescent sera recovered from hosts infected with these attenuated pathogens must contain protective antibodies.

**Figure 2:**
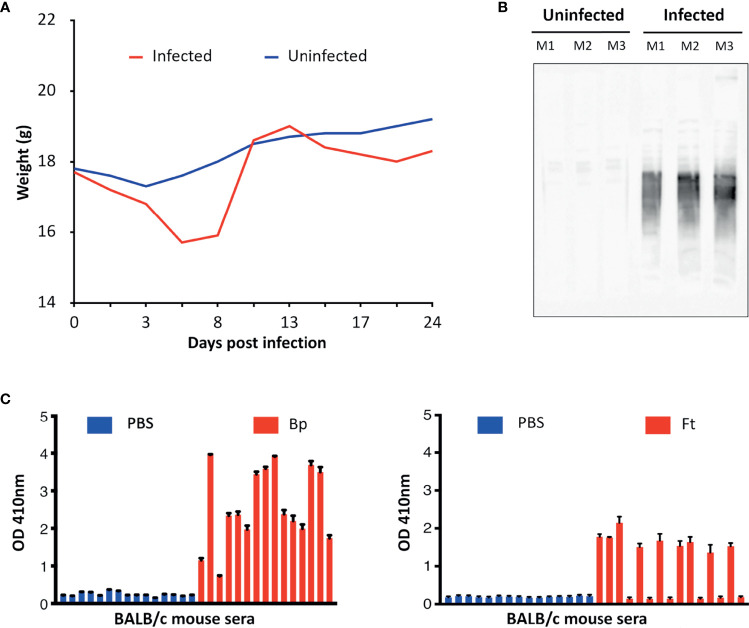
**(A)** Representative course of mouse infection to obtain immune sera. Mice were infected with a sublethal dose of Bp and their weight monitored. Weight was monitored throughout the course of infection. **(B)** Representative Western blot of sera from infected *vs* uninfected mice. Bp protein lysates were analyzed by Western blotting using sera from infected and uninfected mice (Mouse 1–3) and bound antibodies detected using anti-mouse HRP. **(C)** Representative ELISA results obtained from mice infected with Bp and Ft (red) in comparison with uninfected mice (PBS-treated mice, blue). Seroreactivity of mice sera to microwells coated with corresponding pathogen lysate was assessed using protein-A/G-HRP and measuring sample absorbance (optical density). Sera of some mice infected with Ft did not yield positive results because Ft infection led to lethal outcome and mice had to be euthanized during the course of immunization. Graphs represent two technical replicates for sera collected from each mouse. Antibodies from sera with the strongest Western blot and ELISA signals were purified in this study and used to screen for immunogenic peptides.

Briefly, proteins purified from pathogen lysates were digested with trypsin to generate a peptide library. Mice were infected with a sublethal dose of Ft-ΔclpB or Bp82, and immune status assessed through observed weight loss and measurement of seroreactivity of mouse sera to pathogen lysates *via* enzyme-linked immunosorbent assay (ELISA) or Western blot analysis (**Figure 2**). Antibodies purified from the convalescent sera of infected mice were immobilized on magnetic beads and then incubated with pathogen-derived peptides to allow formation of antigen-antibody complexes. Peptides recovered from the immobilized antibodies were identified *via* liquid chromatography coupled with tandem mass spectrometry.

### Bioinformatic Identification of Enriched Antigenic Peptides

The peptides recovered using pooled sera from infected mice (Experiment peptidome) were compared to those recovered from mock-infected mice (Control peptidome); a total of 8 pairs of Experiment-Control peptidomes were collected for Ft, and 9 pairs for Bp. For Ft, we found that out of the 1923 peptides that were recovered in at least two Experiment peptidomes, 44 had an enrichment score of 6 or greater (**Table 1**), whereas only 20.1 +/- 6.1 peptides would be expected at random (p=1x10^-6^). For Bp, out of 2902 peptides that were recovered in at least two Experiment peptidomes, 46 peptides had an enrichment score of 6 or greater (**Table 2**), whereas only 17.8 +/- 4.3 peptides would be expected at random (p=1.9x10^-9^). If a more stringent enrichment cutoff is desired, we found 16 Ft peptides with an enrichment score of 7 or greater, *versus* 3.5+/-1.6 expected at random (p=1.8x10^-7^), and 20 Bp peptides with an enrichment score of 7 or greater, *versus* 4.1+/-2.1 expected at random (p=3.9x10^-9^). The enriched peptides included some that were derived from protective antigens identified in previous studies, as well as predicted outer membrane and extracellular proteins (**Tables 1**, **2**). There were many examples of multiple enriched peptides originating from the same protein (highlighted in bold in the tables), a further indication that enrichment was not random but rather due to immune response to a discrete set of bacterial proteins.

Note that we used C57BL/6J mice for two of the eight Ft experimental samples, because of previously reported differences in protection and antibody response after immunization of C57BL/6J and BALB/c mice with Ft-ΔclpB by Twine et al. (38). Analyzing the BALB/c Ft samples separately yielded a very similar set of results as in **Table 1**, but with lower p-value for the enrichment due to the smaller number of samples (data not shown). Therefore, we decided to combine the data and focus on antibody responses in common between both strains of mice. Although Twine et al. reported an antibody response against chaperonin protein GroL only in BALB/c mice, our data shows that there are several GroL epitopes that are enriched in samples from both mouse strains (see **Table 1** and **Figure 4**).

Prior immunoproteomics analysis of the antibody response to *F. tularensis* using human or mouse sera has identified 164 antibody targets out of a total of 1667 proteins (~10% of the entire Ft proteome) (19, 27, 28). Out of the 1923 peptides that have hits in at least two Ft datasets, 876 peptides match known antigenic proteins. Given those numbers, we would expect only 20 such peptides to show up at random in our list of 44 in **Table 1**, but instead we observe that 38/44 peptides in the list correspond to known antigens - an almost two-fold enrichment (p=2.79x10^-9^). Note that despite the extensive literature on antigens in Ft, only five B-cell epitopes have been experimentally determined (**Figure 4B**), justifying the need for a simple experimental epitope screening method. The immune response to *B. pseudomallei* has not been studied in as much depth as for *Francisella*. So even though Bp with 6203 protein coding genes has a genome that is more than three times as large as that of Ft, we found only 61 known antigens identified in previous studies (29, 30) (~1% of the entire proteome). Our list of 46 top Bp peptides in **Table 1** includes one known antigen, which does not qualify as a statistically significant enrichment primarily because of the much smaller total number of known antigens for Bp.

**Figure 3:**
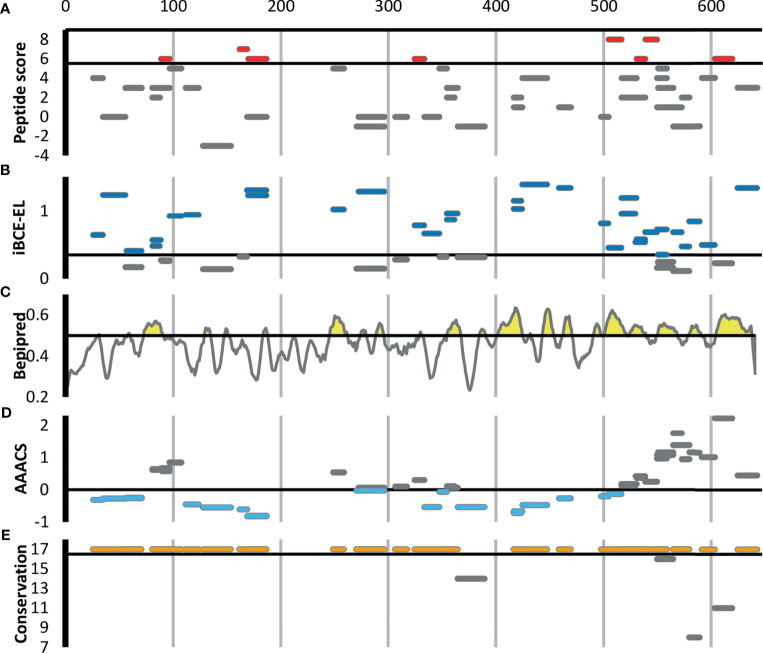
Scoring for the 46 *F. tularensis* DnaK peptides detected in at least two Experiment samples. The short horizontal line segments in A, B, E and F indicate the position of a peptide along the length of the 642aa DnaK protein, and its vertical position within each figure panel indicates its score for the metric indicated. The default score threshold for each tool is shown with a horizontal line, and the peptides or per-amino acid scores exceeding that threshold are shown in color. **(A)** Peptide enrichment score based on our proteomics results. An enrichment score of 8 indicates that the peptide was detected in greater abundance in all 8 Experiment samples relative to their respective Control samples. The threshold for inclusion in **Table 1** was an enrichment score of ≥6 (shown in red). **(B)** B-cell epitope prediction score generated using iBCE-EL. At the default iBCE-EL score threshold of 0.35, nearly three quarter of all peptides were predicted to be likely B-cell epitopes (shown in dark blue). **(C)** B-cell epitope prediction score generated using Bepipred 2.0. The per-amino acid scores are indicated by the line graph. At the default iBCE-EL score threshold of 0.35, 37% of all amino acids were predicted to be in B-cell epitopes (regions of the graph shown in yellow). **(D)** Average Amino Acid Conservation Score (AAACS) based on Consurf analysis. Negative scores indicate greater degrees of evolutionary conservation (shown in light blue). **(E)** Number of fully sequenced *F. tularensis* subsp. tularensis genomes (17 analyzed) in which each peptide occurs. Almost all of the DnaK peptides considered were conserved in all 17 Ft genomes (shown in orange).

**Figure 3** shows all 46 Ft DnaK peptides that were detected in at least two Experiment samples, regardless of their degree of enrichment. Eight of these DnaK peptides are in our list of 44 enriched Ft peptides (**Table 1** and red line segments in **Figure 3A**), including two that are enriched in all 8 Experiments (red line segments in **Figure 3A**). Note the lack of correlation between our experimental enrichment scores and the iBCE-EL and Bepipred scores (**Figures 3B, C**). All but one of the 8 enriched peptides are conserved in all 17 fully sequenced Ft strains (**Figure 3E**), but some of the peptides towards the C-terminal show a greater evolutionary rate as measured by their Average Amino Acid conservation Score (AAACS, **Figure 3D**) and thus may be more prone to immune escape mutants.

**Figure 4:**
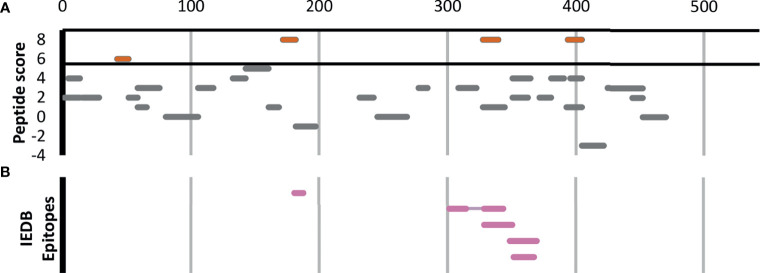
The 32 *F. tularensis* GroL peptides detected in at least two Experiment samples. Horizontal line segments indicate the position of each peptide along the length of the 544aa GroL protein sequence. **(A)** Peptide enrichment score based on our proteomics results, with a score of 8 indicating that the peptide was found in greater abundance in all 8 Experiment samples relative to their respective Control samples. The threshold for inclusion in **Table 1** was a score of ≥6 or better (shown in red). **(B)** Five B-cell epitopes identified by DXMS by Lu et al. (39), including one discontinuous epitope.

**Figure 4** shows all 32 Ft GroL peptides that were detected in at least two Experiment samples in our study, regardless of the degree of their enrichment. Four of these GroL peptides are in our list of 44 enriched Ft peptides (**Table 1** and red line segments in **Figure 4A**), including three that are enriched in all eight Experiments. Lu et al. (39) used hydrogen/deuterium exchange–mass spectrometry (DXMS) to experimentally identify one discontinuous and four linear B-cell epitopes for a selection of mouse monoclonal antibodies against GroL (**Figure 4B**). Note that one of the four enriched peptides in **Figure 4A** (DNTTIIDGAGEK) overlaps with a linear epitope (NTTIIDGAGEKEAIAKRINVIK) and a discontinuous epitope (SEDLSMKLEETNM—NTTIIDGAGEKEAIA) identified by DXMS in **Figure 4B**, while a second enriched peptide (EGVITVEEGK) is directly adjacent to another of the linear DXMS epitopes (FEDEL). According to the Immune Epitope Database (IEDB) (40), these are the only experimentally validated B-cell epitopes for Ft (IEDB also lists four B. pseudomallei antigens that have been assayed for B-cell epitopes, none of which overlap with the proteins in **Table 1**).

## Discussion

We have developed a widely applicable shotgun immunoproteomic method that enables efficient identification of B-cell epitopes in the proteomes of pathogens. The results of this study have revealed a significant enrichment of peptides derived from previously identified antigens and vaccine candidates, validating the method’s efficacy. This method was designed to identify linear epitopes efficiently without the need of genetic manipulation or other experimental techniques that can be costly and labor intensive. Attenuated strains made the optimization of this proof-of-concept study more efficient; however, the availability of an attenuated strain for the target organism does not represent a limitation, as our strategy could be applied to fully virulent strains of pathogens as well. Although the present study was performed using a mouse model, the workflow could be easily adapted to detecting targets relevant to the human immune system, using convalescent sera from patients.

Utilizing peptide antigens for vaccine development has several advantages over typical vaccine development efforts. Similar to other types of subunit vaccines, peptide vaccines represent a safer alternative to attenuated vaccines due to lack of any potentially infectious materials in the vaccine formulation. Use of short peptides sufficient for stimulation of immune response favors exclusion of deleterious sequences that may be present in full length antigenic proteins. Peptide vaccine formulations are defined and their contents fully synthetic, which simplifies quality control procedures and thereby streamlines the regulatory approval process. Production of peptide vaccines is expected to be relatively fast and inexpensive, due to ease of synthesis and recent advances in improved peptide stability (3, 41, 42). Moreover, once antigenic peptides are identified, evaluation of their efficacy could represent a lesser challenge due to the possibility of multiplexing peptides during *in vivo* trials, rather than use of one-at-a-time testing

Among Ft proteins, the present screen identified multiple peptides for two well-characterized antigens, 60kDa chaperonin GroL (Q5NEE1) and chaperone protein DnaK (Q5NFG7). Both chaperonins have been previously implicated in virulence of Francisella (43–45), and are known to induce antibody production in mice and humans (27, 46, 47). These chaperonin proteins are important for facilitating folding of nascent proteins as well as post-translational modifications. They are also known as heat-shock proteins, as they protect cellular proteins from environmental stresses such as high temperature and low pH (47, 48). Although their cellular localization is predicted to be cytoplasmic, they reportedly also associate with membrane proteins and are released into host cells during infection (47, 49–51) perhaps contributing to their ability to stimulate various immune functions, including innate immunity, humoral immunity and cell-mediated immunity (43, 47, 52–55). Heat-shock proteins are good candidates for subunit vaccine design due to their ability to stimulate various immune responses without the need of adjuvant; in fact, both GroL and DnaK have been exploited for vaccine development efforts targeting *Francisella* and other pathogens (39, 47, 56, 57).

Highly virulent Type A *Francisella* strains such as SCHU S4 can bind host plasminogen to the bacterial cell surface where it can be converted to plasmin, a serine protease that degrades opsonizing antibodies, inhibiting antibody-mediated uptake by macrophages (58, 59). Among the 25 Ft proteins listed in **Table 1**, we find at least 3 that are known to be involved in plasminogen binding in *Francisella* or other pathogens, including conserved hypothetical lipoprotein LpnA (Q5NGE4) (59), fructose-1,6-bisphosphate aldolase (Q5NF78) (60), and elongation factor Tu (Q5NID9) (61). These proteins could make for particularly attractive vaccine targets, because if we can interfere with their function before the pathogen has activated its plasmin-mediated antibody evasion, that would make it more susceptible to other antibodies as well.

Among the antigenic peptides identified in the Bp proteome are those belonging to Type VI secretion system component Hcp-,1 and previously identified antigen 10kDa chaperonin GroES (62). Hcp-1 was previously found to be a major virulence determinant in *Burkholderia* and recognized by sera from infected human patients and animals (63–65). Due to this, Hcp-1 has been interrogated as a potential candidate for *Burkholderia* vaccine development (63–65). Additionally, a peptide from an ankyrin repeat-containing protein (A0A0H3HJC) came up as one of the highest scoring peptides in our study. Ankyrin repeats are typically eukaryotic protein domains involved in protein-protein interactions (66), but have been co-opted by many bacterial pathogens as type IV secreted effector proteins to mimic or manipulate various host functions (67).

Recovery of peptides derived from several supposedly cytosolic enzymes may seem puzzling. However several “housekeeping” enzymes are known to be displayed on the surface of pathogens where they play a role in virulence (68). For example, our top scoring peptides from *B. pseudomallei* include two derived from enolase (A0A0H3HLA6). While enolase is primarily thought of as a key glycolytic enzyme, it is also expressed on the surface of a wide variety of bacterial and fungal pathogens, where it interacts with host plasminogen and is associated with invasion and virulence (69). Antibodies against enolase have been detected in a large variety of infectious and autoimmune diseases (70). It is as yet unknown whether enolase plays the same role in Burkholderia, but the protein is predicted to be present both in the cytoplasm and on the cell surface, and its production was found to be upregulated upon exposure to human lung epithelial cells (71). Other housekeeping proteins in our top scoring results whose homologs in other pathogens are known to play a role in adhesion, invasion, or virulence include elongation factor Tu (Q5NID9), malic enzyme/malate dehydrogenase (A0A0H3HP28, Q5NHC8), and fructose-1,6-bisphosphate aldolase (Q5NF78) (68).

Overall, this immunoproteomic workflow has identified numerous peptides mapping to previously identified antigens and subunit vaccine targets, predicted membrane-associated proteins, as well as uncharacterized proteins. The Ft datasets revealed a significant enrichment of peptides belonging to previously identified antigenic proteins in Experiment samples relative to their respective Control samples, providing validation to this approach. Interestingly, several of these known antigens also yielded multiple top scoring peptides in our analysis. Despite the large amount of prior immunoproteomic analysis on Ft, covering ~10% of the genome, experimentally validated B-cell epitopes are available for only a single protein, and our analysis captures two out of its five known epitopes. Due to the much smaller number of previously identified antigens for Burkholderia, we were not able to tell whether the enrichment in the Bp datasets was significant. Improved proteome coverage and more comprehensive immunogenic profiles could be achieved with the use of alternative enzymes with different specificities, since there is a risk of ablating epitopes that contain cut sites recognized by specific enzymes such as trypsin. Alternatively, performing incomplete digestion with one enzyme, or a cocktail of enzymes with different specificities, could increase the number of overlapping peptides and thereby improve the yield and diversity of identified epitopes. In addition, since the presented method is dependent upon extraction of proteins from whole cell lysates, it is conceivable that the proteome coverage could be biased toward highly abundant proteins or those proteins that are easier to extract, despite this disadvantage we have detected several membrane-bound antigens in this study.

A variety of computational B-cell epitope prediction tools have been developed to identify epitopes in antigens. However accurate computational prediction of B-cell epitopes still poses a major challenge (72), with sensitivity or specificity typically below 60% (35, 73–76), leading some recent in-silico multi-epitope vaccine design efforts to look at the consensus of up to 8 or 9 B-cell epitope prediction tools simultaneously (77, 78). The recent development of prediction tools using state-of-the-art machine learning models that claim significantly higher performance on large benchmarking datasets seems promising (34, 79). Here we compare the performance of Bepipred 2.0 (35), one of the most widely used B-cell prediction tools, and iBCE-EL (34). Interestingly, we find no significant correlation between the peptides experimentally identified using the method described here and computationally predicted linear B-cell epitope scores generated by Bepipred 2.0 and iBCE-EL, even for those antibody-binding peptides belonging to known Ft or Bp antigens, nor do we find any significant correlation between the Bepipred 2.0 and iBCE-EL scores themselves (see **Supplementary Tables S1, S2**, as well as **Figures 3A–C** for Ft DnaK), highlighting the value of an unbiased experimental method to screen for antibody targets, as presented here. At their default score thresholds, iBCE-EL correctly predicts 34/44 of the Ft peptides, and 39/46 of the Bp peptides, while Bepipred 2.0 correctly predicts 21/44 Ft peptides and 13/46 Bp peptides, but that is not actually significantly more than would be expected at random given their hit rates on other un-enriched tryptic peptides in our dataset. Part of the discrepancy between the computational Bepipred 2.0 predictions and our experimental results may be due to the fact that Bepipred 2.0 is trained on antigen-antibody 3D structures, which likely contain a mix of conformational and linear epitopes. In addition, Bepipred 2.0 has a relatively low self-reported 58.6% sensitivity and 57.2% specificity at the default score threshold of 0.5 (80), and thus is expected to exhibit a large number of false positives and false negative predictions. iBCE-EL is reported to have better sensitivity and specificity [73.2% and 72.4% (34)], but explicitly takes into account sequence features at the beginning and end of the epitope that may be missing in the tryptic peptides generated here, affecting their score. In cases where the tryptic peptide is too short to be used directly as a vaccine candidate (some are as short as 6 residues), we may in fact be able to use these computational tools to guide us in how to extend the boundaries of the peptide beyond its flanking trypsin cleavage sites.

Note that computational B-cell prediction tools such as these are trained to distinguish epitopes from non-epitopes in known antigens, but are not an effective alternative to experimentally screening for epitopes across an entire bacterial proteome. For example, on a random selection of 100 Ft and Bp proteins, Bepipred-2.0 using its default epitope threshold of 0.5 classified 40% of all amino acids as being part of an epitope, including an average of 5.5 peptides of length 9 or longer per protein (data not shown). Likewise, on a random selection of 1000 tryptic peptides from all our proteomics data, iBCE-EL classified 81% as B-cell epitopes using its default score threshold of 0.35 (data not shown). Applied across the entire proteome, the computational approach would predict tens of thousands of putative B-cell epitopes, likely with a high false-positive rate and, regardless, providing little guidance in winnowing the possibilities for experimental verification.

If so desired, peptides can be downselected for vaccine development by focusing only on those with the most stringent enrichment scores, or based on consensus with computational epitope prediction tools. Further downselection may include prioritizing highly conserved epitopes that can induce broadly protective immunity, and reduce the risk that emergence of pathogen variants will render the vaccine ineffective (81). ~90% of the top scoring peptides were found to be present in 90% or more of the fully sequenced pathogenic F. tularensis and B. pseudomallei strains (see **Supplementary Tables S1, S2**, and **Figure 3F** for the case of Ft DnaK). In addition, we can target peptides that show even deeper evolutionary conservation based on their Average Amino Acid Conservation Score (AAACS), reflecting parts of the protein that may be important for its function (31) (see **Supplementary Tables S1, S2**, and **Figure 3E** for the case of Ft DnaK). Peptides that are only one or two amino acids different from human or mouse versions are likely less suitable as vaccine candidates and are marked with a subscript 1 or 2 respectively in **Tables 1**, **2**. Note that while some of the proteins in **Tables 1**, **2** have homologs in human and mouse (e.g. mitochondrial DnaK), the peptides recovered here are unique to the bacterial versions. For vaccine design, we may also want to prioritize peptides which do not tend to occur in healthy human microbiomes, by comparing them against some of the large human metaproteomics datasets recently generated (82–86).

Further confirmation that the identified sequences are B-cell epitopes could be achieved through additional *in vitro* and *in vivo* experimentation (e.g., testing the reactivity of immune sera with synthesized candidate epitopes *via* ELISA or immunization studies). High throughput screening of peptides for efficacy is feasible due to recent advancements in solid phase peptide synthesis (SPPS), which enables efficient and cost-effective production of peptide candidates (3). For immunization studies, pools of multiple peptides could be incorporated into vaccine delivery systems containing adjuvants and T-helper epitopes known to stimulate the induction of adaptive immune response against peptide antigens, as reviewed in Skwarczynski et al. (3).

The method presented here identifies peptides that are immunoreactive, that is, they interact with antibodies in serum from previously infected individuals. Further experimental test would be needed to confirm immunogenicity, that is, whether they can stimulate antibody production themselves, and protectivity, that is, whether they can protect against infection or disease after immunization. Our immunoproteomic method represents a new tool for precise mapping of linear B-cell epitopes. Generation of such immunogenic profiles for pathogens could provide an ample pool of candidates for further experimental validation and efficient vaccine development. Accelerating the discovery of B-cell epitopes in the proteomes of pathogens will help fuel the development of peptide-based vaccines that have the potential to provide rapid solutions to biothreat agents and emerging pathogens.

## Data Availability Statement

The datasets presented in this study can be found in online repositories. The names of the repository/repositories and accession number(s) can be found below: https://www.ebi.ac.uk/pride/archive/, PXD026300 (87).

## Ethics Statement

The animal study was reviewed and approved by LLNL Institutional Animal Care and Use Committee.

## Author Contributions

PD’h, NC, and MF contributed to the conception and design of the study. NC performed the *in vivo* experiments. VL provided laboratory support. VL and MF performed *in vitro* experimentation. PD performed the bioinformatics analysis. BS and SB provided critical input. All authors contributed to the article and approved the submitted version.

## Funding

This work was supported by Lawrence Livermore National Laboratory Directed Research and Development Program (LLNL LDRD) Labwide grant (18-LW-039) to MF, and by the LDRD program (grant 218309) at Sandia National Laboratories, a multi-mission laboratory managed and operated by National Technology and Engineering Solutions of Sandia, LLC, a wholly owned subsidiary of Honeywell International, Inc., for the U.S. Department of Energy’s National Nuclear Security Administration under contract DE-NA0003525. Work at LLNL was performed under the auspices of the U.S. Department of Energy by Lawrence Livermore National Laboratory under Contract DE-AC52-07NA27344. LLNL IM release number LLNL-JRNL-822446. The funders were not involved in the study design, collection, analysis, interpretation of data, the writing of this article or the decision to submit it for publication.

## Conflict of Interest

MF, NC, and PD’h are inventors on a provisional patent application for the method for rapid detection of immunogenic epitopes, filed by Lawrence Livermore National Security, LLC.

The remaining authors declare that the research was conducted in the absence of any commercial or financial relationships that could be construed as a potential conflict of interest.

## Publisher’s Note

All claims expressed in this article are solely those of the authors and do not necessarily represent those of their affiliated organizations, or those of the publisher, the editors and the reviewers. Any product that may be evaluated in this article, or claim that may be made by its manufacturer, is not guaranteed or endorsed by the publisher.

## References

[1] Li W Joshi MD Singhania S Ramsey KH Murthy AK. Peptide Vaccine: Progress and Challenges. Vaccines Basel (2014) 2(3):515–36. doi: 10.3390/vaccines2030515 PMC4494216 26344743

[2] Malonis RJ Lai JR Vergnolle O. Peptide-Based Vaccines: Current Progress and Future Challenges. Chem Rev (2020) 120(6):3210–29. doi: 10.1021/acs.chemrev.9b00472 PMC7094793 31804810

[3] Skwarczynski M Toth I. Peptide-Based Synthetic Vaccines. Chem Sci (2016) 7(2):842–54. doi: 10.1039/C5SC03892H PMC5529997 28791117

[4] Sun P Ju H Liu Z Ning Q Zhang J Zhao X. Bioinformatics Resources and Tools for Conformational B-Cell Epitope Prediction. Comput Math Methods Med (2013) 2013:943636. doi: 10.1155/2013/943636 23970944 PMC3736542

[5] Dudek NL Perlmutter P Aguilar MI Croft NP Purcell AW. Epitope Discovery and Their Use in Peptide Based Vaccines. Curr Pharm Des (2010) 16(28):3149–57. doi: 10.2174/138161210793292447 20687873

[6] Wang AP Li N Zhou JM Chen YM Jiang M Qi YH. Mapping the B Cell Epitopes Within the Major Capsid Protein L1 of Human Papillomavirus Type 16. Int J Biol Macromol (2018) 118:1354–61. doi: 10.1016/j.ijbiomac.2018.06.094 29959021

[7] Zhao DM Han KK Huang XM Zhang LJ Wang HL Liu N. Screening and Identification of B-Cell Epitopes Within Envelope Protein of Tembusu Virus. Virol J (2018) 15:142. doi: 10.1186/s12985-018-1052-1 30223850 PMC6142368

[8] Bi Y Jin Z Wang Y Mou S Wang W Wei Q. Identification of Two Distinct Linear B Cell Epitopes of the Matrix Protein of the Newcastle Disease Virus Vaccine Strain LaSota. Viral Immunol (2019) 32(5):221–9. doi: 10.1089/vim.2019.0007 31094659

[9] Jaenisch T Heiss K Fischer N Geiger C Bischoff FR Moldenhauer G. High-Density Peptide Arrays Help to Identify Linear Immunogenic B-Cell Epitopes in Individuals Naturally Exposed to Malaria Infection. Mol Cell Proteomics (2019) 18(4):642–56. doi: 10.1074/mcp.RA118.000992 PMC6442360 30630936

[10] Yang W-J Lai J-F Peng K-C Chiang H-J Weng C-N Shiuan D. Epitope Mapping of Mycoplasma Hyopneumoniae Using Phage Displayed Peptide Libraries and the Immune Responses of the Selected Phagotopes. J Immunol Methods (2005) 304(1–2):15–29. doi: 10.1016/j.jim.2005.05.009 16054642

[11] Mullen LM Nair SP Ward JM Rycroft AN Henderson B. Phage Display in the Study of Infectious Diseases. Trends Microbiol (2006) 14(3):141–7. doi: 10.1016/j.tim.2006.01.006 PMC7127285 16460941

[12] Ndam NT Denoeud-Ndam L Doritchamou J Viwami F Salanti A Nielsen MA. Protective Antibodies Against Placental Malaria and Poor Outcomes During Pregnancy, Benin. Emerg Infect Dis (2015) 21(5):813–23. doi: 10.3201/eid2105.141626 PMC4412227 25898123

[13] Gonzales SJ Reyes RA Braddom AE Batugedara G Bol S Bunnik EM. Naturally Acquired Humoral Immunity Against *Plasmodium Falciparum* Malaria. Front Immunol (2020) 11:594653. doi: 10.3389/fimmu.2020.594653 33193447 PMC7658415

[14] Li H Wang X-X Wang B Fu L Liu G Lu Y. Latently and Uninfected Healthcare Workers Exposed to TB Make Protective Antibodies Against Mycobacterium Tuberculosis. Proc Natl Acad Sci USA (2017) 114(19):5023–8. doi: 10.1073/pnas.1611776114 PMC5441709 28438994

[15] Sharma G Holt RA. T-Cell Epitope Discovery Technologies. Hum Immunol (2014) 75(6):514–9. doi: 10.1016/j.humimm.2014.03.003 24755351

[16] Sharma G Rive CM Holt RA. Rapid Selection and Identification of Functional CD8+ T Cell Epitopes From Large Peptide-Coding Libraries. Nat Commun (2019) 10:4553. doi: 10.1038/s41467-019-12444-7 31591401 PMC6779888

[17] Caron E Kowalewski DJ Chiek Koh C Sturm T Schuster H Aebersold R. Analysis of Major Histocompatibility Complex (MHC) Immunopeptidomes Using Mass Spectrometry. Mol Cell Proteomics MCP (2015) 14(12):3105–17. doi: 10.1074/mcp.O115.052431 PMC4762616 26628741

[18] Dienst FT. Tularemia: A Perusal of Three Hundred Thirty-Nine Cases. J State Med Soc (1963) 115:114–27. 14027775

[19] Fulton KM Zhao X Petit MD Kilmury SLN Wolfraim LA House RV. Immunoproteomic Analysis of the Human Antibody Response to Natural Tularemia Infection With Type A or Type B Strains or LVS Vaccination. Int J Med Microbiol IJMM (2011) 301(7):591–601. doi: 10.1016/j.ijmm.2011.07.002 21873113 PMC3184521

[20] Gibney KB Cheng AC. Reducing the Melioidosis Burden: Public Health, Chronic Disease Prevention, or Improved Case Management? Lancet Infect Dis (2019) 19(8):800–2. doi: 10.1016/S1473-3099(19)30303-2 31285142

[21] Mara-Koosham G Hutt JA Lyons CR Wu TH. Antibodies Contribute to Effective Vaccination Against Respiratory Infection by Type A Francisella Tularensis Strains. Infect Immun (2011) 79(4):1770–8. doi: 10.1128/IAI.00605-10 PMC3067570 21282410

[22] Ray HJ Cong Y Murthy AK Selby DM Klose KE Barker JR. Oral Live Vaccine Strain-Induced Protective Immunity Against Pulmonary Francisella Tularensis Challenge Is Mediated by CD4+ T Cells and Antibodies, Including Immunoglobulin a. Clin Vaccine Immunol CVI (2009) 16(4):444–52. doi: 10.1128/CVI.00405-08 PMC2668291 19211773

[23] Rhinehart-Jones TR Fortier AH Elkins KL. Transfer of Immunity Against Lethal Murine Francisella Infection by Specific Antibody Depends on Host Gamma Interferon and T Cells. Infect Immun (1994) 62(8):3129–37. doi: 10.1128/iai.62.8.3129-3137.1994 PMC302937 8039881

[24] Hogan RJ Lafontaine ER. Antibodies Are Major Drivers of Protection Against Lethal Aerosol Infection With Highly Pathogenic *Burkholderia* spp. mSphere (2019) 4:e00674–18. doi: 10.1128/mSphere.00674-18 PMC6315082 30602525

[25] Jones SM Ellis JF Russell P Griffin KF Oyston PCF. Passive Protection Against *Burkholderia Pseudomallei* Infection in Mice by Monoclonal Antibodies Against Capsular Polysaccharide, Lipopolysaccharide or Proteins. J Med Microbiol (2002) 51(12):1055–62. doi: 10.1099/0022-1317-51-12-1055 12466403

[26] Khakhum N Bharaj P Myers JN Tapia D Kilgore PB Ross BN. *Burkholderia Pseudomallei* ΔtonB Δhcp1 Live Attenuated Vaccine Strain Elicits Full Protective Immunity Against Aerosolized Melioidosis Infection. mSphere (2019) 4:e00670–18. doi: 10.1128/mSphere.00570-18 PMC6315081 30602524

[27] Kilmury SLN Twine SM. The Francisella Tularensis Proteome and its Recognition by Antibodies. Front Microbiol (2010) 1:143. doi: 10.3389/fmicb.2010.00143 21687770 PMC3109489

[28] Nakajima R Escudero R Molina DM Rodríguez-Vargas M Randall A Jasinskas A. Towards Development of Improved Serodiagnostics for Tularemia by Use of Francisella Tularensis Proteome Microarrays. J Clin Microbiol (2016) 54(7):1755–65. doi: 10.1128/JCM.02784-15 PMC4922089 27098957

[29] Felgner PL Kayala MA Vigil A Burk C Nakajima-Sasaki R Pablo J. A *Burkholderia Pseudomallei* Protein Microarray Reveals Serodiagnostic and Cross-Reactive Antigens. Proc Natl Acad Sci U S A (2009) 106(32):13499–504. doi: 10.1073/pnas.0812080106 PMC2717108 19666533

[30] Yi J Simpanya MF Settles EW Shannon AB Hernandez K Pristo L. Caprine Humoral Response to Burkholderia Pseudomallei Antigens During Acute Melioidosis From Aerosol Exposure. PloS Negl Trop Dis (2019) 13(2):e0006851. doi: 10.1371/journal.pntd.0006851 30811382 PMC6411198

[31] Ashkenazy H Abadi S Martz E Chay O Mayrose I Pupko T. ConSurf 2016: An Improved Methodology to Estimate and Visualize Evolutionary Conservation in Macromolecules. Nucleic Acids Res (2016) 44(W1):W344–50. doi: 10.1093/nar/gkw408 PMC4987940 27166375

[32] Ren J Ellis J Li J. Influenza A Ha’s Conserved Epitopes and Broadly Neutralizing Antibodies: A Prediction Method. J Bioinform Comput Biol (2014) 12(5):1450023. doi: 10.1142/S0219720014500231 25208658

[33] Fiuza TS Lima JPMS de Souza GA. EpitoCore: Mining Conserved Epitope Vaccine Candidates in the Core Proteome of Multiple Bacteria Strains. Front Immunol (2020) 11:816. doi: 10.3389/fimmu.2020.00816 32431712 PMC7214623

[34] Manavalan B Govindaraj RG Shin TH Kim MO Lee G. iBCE-EL: A New Ensemble Learning Framework for Improved Linear B-Cell Epitope Prediction. Front Immunol (2018) 9:1695. doi: 10.3389/fimmu.2018.01695 30100904 PMC6072840

[35] Jespersen MC Peters B Nielsen M Marcatili P. BepiPred-2.0: Improving Sequence-Based B-Cell Epitope Prediction Using Conformational Epitopes. Nucleic Acids Res (2017) 45(W1):W24–9. doi: 10.1093/nar/gkx346 PMC5570230 28472356

[36] Conlan JW Shen H Golovliov I Zingmark C Oyston PC Chen W. Differential Ability of Novel Attenuated Targeted Deletion Mutants of Francisella Tularensis Subspecies Tularensis Strain SCHU S4 to Protect Mice Against Aerosol Challenge With Virulent Bacteria: Effects of Host Background and Route of Immunization. Vaccine (2010) 28(7):1824–31. doi: 10.1016/j.vaccine.2009.12.001 PMC2822029 20018266

[37] Propst KL Mima T Choi KH Dow SW Schweizer HP. A Burkholderia Pseudomallei deltapurM Mutant is Avirulent in Immunocompetent and Immunodeficient Animals: Candidate Strain for Exclusion From Select-Agent Lists. Infect Immun (2010) 78(7):3136–43. doi: 10.1128/IAI.01313-09 PMC2897367 20404077

[38] Twine S Shen H Harris G Chen W Sjostedt A Ryden P. BALB/c Mice, But Not C57BL/6 Mice Immunized With a ΔclpB Mutant of Francisella tularensis Subspecies Tularensis are Protected Against Respiratory Challenge With Wild-Type Bacteria: Association of Protection With Post-Vaccination and Post-Challenge Immune Responses. Vaccine (2012) 30(24):3634–45. doi: 10.1016/j.vaccine.2012.03.036 22484348

[39] Lu Z Rynkiewicz MJ Madico G Li S Yang CY Perkins HM. B-Cell Epitopes in GroEL of Francisella tularensis. PloS One (2014) 9(6):e99847. doi: 10.1371/journal.pone.0099847 24968190 PMC4072690

[40] Vita R Mahajan S Overton JA Dhanda SK Martini S Cantrell JR. The Immune Epitope Database (IEDB): 2018 Update. Nucleic Acids Res (2019) 47(D1):D339–43. doi: 10.1093/nar/gky1006 PMC6324067 30357391

[41] Purcell AW McCluskey J Rossjohn J. More Than One Reason to Rethink the Use of Peptides in Vaccine Design. Nat Rev Drug Discovery (2007) 6(5):404–14. doi: 10.1038/nrd2224 17473845

[42] Sun T Han H Hudalla GA Wen Y Pompano RR Collier JH. Thermal Stability of Self-Assembled Peptide Vaccine Materials. Acta Biomater (2016) 30:62–71. doi: 10.1016/j.actbio.2015.11.019 26584836 PMC4821069

[43] Noah CE Malik M Bublitz DC Camenares D Sellati TJ Benach JL. GroEL and Lipopolysaccharide From Francisella Tularensis Live Vaccine Strain Synergistically Activate Human Macrophages. Infect Immun (2010) 78(4):1797–806. doi: 10.1128/IAI.01135-09 PMC2849404 20123721

[44] Pechous RD McCarthy TR Zahrt TC. Working Toward the Future: Insights Into Francisella tularensis Pathogenesis and Vaccine Development. Microbiol Mol Biol Rev (2009) 73(4):684–711. doi: 10.1128/MMBR.00028-09 19946137 PMC2786580

[45] Weiss DS Brotcke A Henry T Margolis JJ Chan K Monack DM. *In Vivo* Negative Selection Screen Identifies Genes Required for Francisella Virulence. Proc Natl Acad Sci USA (2007) 104(14):6037–42. doi: 10.1073/pnas.0609675104 PMC1832217 17389372

[46] Havlasova J Hernychova L Brychta M Hubalek M Lenco J Larsson P. Proteomic Analysis of Anti-Francisella Tularensis LVS Antibody Response in Murine Model of Tularemia. Proteomics (2005) 5(8):2090–103. doi: 10.1002/pmic.200401123 15892173

[47] Huntley JF Conley PG Hagman KE Norgard MV. Characterization of Francisella tularensis Outer Membrane Proteins. J Bacteriol (2007) 189(2):561–74. doi: 10.1128/JB.01505-06 PMC1797401 17114266

[48] Ericsson M Tarnvik A Kuoppa K Sandstrom G Sjostedt A. Increased Synthesis of DnaK, GroEL, and GroES Homologs by Francisella Tularensis LVS in Response to Heat and Hydrogen Peroxide. Infect Immun (1994) 62(1):178–83. doi: 10.1128/iai.62.1.178-183.1994 PMC186084 7903283

[49] Henderson B Allan E Coates AR. Stress Wars: The Direct Role of Host and Bacterial Molecular Chaperones in Bacterial Infection. Infect Immun (2006) 74(7):3693–706. doi: 10.1128/IAI.01882-05 PMC1489680 16790742

[50] Hickey TB Thorson LM Speert DP Daffe M Stokes RW. Mycobacterium Tuberculosis Cpn60.2 and DnaK are Located on the Bacterial Surface, Where Cpn60.2 Facilitates Efficient Bacterial Association With Macrophages. Infect Immun (2009) 77(8):3389–401. doi: 10.1128/IAI.00143-09 PMC2715658 19470749

[51] Lee BY Horwitz MA Clemens DL. Identification, Recombinant Expression, Immunolocalization in Macrophages, and T-Cell Responsiveness of the Major Extracellular Proteins of Francisella tularensis. Infect Immun (2006) 74(7):4002–13. doi: 10.1128/IAI.00257-06 PMC1489726 16790773

[52] Ashtekar AR Zhang P Katz J Deivanayagam CC Rallabhandi P Vogel SN. TLR4-Mediated Activation of Dendritic Cells by the Heat Shock Protein DnaK From Francisella tularensis. J Leukoc Biol (2008) 84(6):1434–46. doi: 10.1189/jlb.0308215 PMC2614597 18708593

[53] Kol A Bourcier T Lichtman AH Libby P. Chlamydial and Human Heat Shock Protein 60s Activate Human Vascular Endothelium, Smooth Muscle Cells, and Macrophages. J Clin Invest (1999) 103(4):571–7. doi: 10.1172/JCI5310 PMC408102 10021466

[54] Wallin RP Lundqvist A More SH von Bonin A Kiessling R Ljunggren HG. Heat-Shock Proteins as Activators of the Innate Immune System. Trends Immunol (2002) 23(3):130–5. doi: 10.1016/S1471-4906(01)02168-8 11864840

[55] Valentino MD Maben ZJ Hensley LL Woolard MD Kawula TH Frelinger JA. Identification of T-Cell Epitopes in Francisella Tularensis Using an Ordered Protein Array of Serological Targets. Immunology (2011) 132(3):348–60. doi: 10.1111/j.1365-2567.2010.03387.x PMC3044901 21214540

[56] Ashtekar AR Katz J Xu Q Michalek SM. A Mucosal Subunit Vaccine Protects Against Lethal Respiratory Infection With Francisella tularensis LVS. PloS One (2012) 7(11):e50460. doi: 10.1371/journal.pone.0050460 23209745 PMC3508931

[57] Khan MN Shukla D Bansal A Mustoori S Ilavazhagan G. Immunogenicity and Protective Efficacy of GroEL (Hsp60) of Streptococcus Pneumoniae Against Lethal Infection in Mice. FEMS Immunol Med Microbiol (2009) 56(1):56–62. doi: 10.1111/j.1574-695X.2009.00548.x 19484809

[58] Crane DD Warner SL Bosio CM. A Novel Role for Plasmin Mediated Degradation of Opsonizing Antibody in the Evasion of Host Immunity by Virulent, But Not Attenuated, Francisella tularensis. J Immunol Baltim Md 1950 (2009) 183(7):4593–600. doi: 10.4049/jimmunol.0901655 PMC2748154 19752236

[59] Clinton SR Bina JE Hatch TP Whitt MA Miller MA. Binding and Activation of Host Plasminogen on the Surface of Francisella tularensis. BMC Microbiol (2010) 10:76. doi: 10.1186/1471-2180-10-76 20226053 PMC2848021

[60] Shams F Oldfield NJ Wooldridge KG Turner DPJ. Fructose-1,6-Bisphosphate Aldolase (FBA)-A Conserved Glycolytic Enzyme With Virulence Functions in Bacteria: “Ill Met by Moonlight.” Biochem Soc Trans (2014) 42(6):1792–5. doi: 10.1042/BST20140203 25399608

[61] Kunert A Losse J Gruszin C Hühn M Kaendler K Mikkat S. Immune Evasion of the Human Pathogen Pseudomonas Aeruginosa: Elongation Factor Tuf is a Factor H and Plasminogen Binding Protein. J Immunol Baltim Md 1950 (2007) 179(5):2979–88. doi: 10.4049/jimmunol.179.5.2979 17709513

[62] Varga JJ Vigil A DeShazer D Waag DM Felgner P Goldberg JB. Distinct Human Antibody Response to the Biological Warfare Agent Burkholderia mallei. Virulence (2012) 3(6):510–4. doi: 10.4161/viru.22056 PMC3524150 23076276

[63] Burtnick MN Brett PJ Harding SV Ngugi SA Ribot WJ Chantratita N. The Cluster 1 Type VI Secretion System Is a Major Virulence Determinant in Burkholderia pseudomallei. Infect Immun (2011) 79(4):1512–25. doi: 10.1128/IAI.01218-10 PMC3067527 21300775

[64] Schell MA Ulrich RL Ribot WJ Brueggemann EE Hines HB Chen D. Type VI Secretion is a Major Virulence Determinant in Burkholderia mallei. Mol Microbiol (2007) 64(6):1466–85. doi: 10.1111/j.1365-2958.2007.05734.x 17555434

[65] Whitlock GC Deeraksa A Qazi O Judy BM Taylor K Propst KL. Protective Response to Subunit Vaccination Against Intranasal Burkholderia mallei and B. pseudomallei Challenge. Proc Vaccinol (2010) 2:73–7. doi: 10.1016/j.provac.2010.03.013 PMC3874274 24379895

[66] Li J Mahajan A Tsai M-D. Ankyrin Repeat: A Unique Motif Mediating Protein-Protein Interactions. Biochemistry (2006) 45(51):15168–78. doi: 10.1021/bi062188q 17176038

[67] Pan X Lührmann A Satoh A Laskowski-Arce MA Roy CR. Ankyrin Repeat Proteins Comprise a Diverse Family of Bacterial Type IV Effectors. Science (2008) 320(5883):1651–4. doi: 10.1126/science.1158160 PMC2514061 18566289

[68] Pancholi V Chhatwal GS. Housekeeping Enzymes as Virulence Factors for Pathogens. Int J Med Microbiol IJMM (2003) 293(6):391–401. doi: 10.1078/1438-4221-00283 14760970

[69] Lottenberg R Minning-Wenz D Boyle MD. Capturing Host Plasmin(Ogen): A Common Mechanism for Invasive Pathogens? Trends Microbiol (1994) 2(1):20–4. doi: 10.1016/0966-842X(94)90340-9 8162432

[70] Terrier B Degand N Guilpain P Servettaz A Guillevin L Mouthon L. Alpha-Enolase: A Target of Antibodies in Infectious and Autoimmune Diseases. Autoimmun Rev (2007) 6(3):176–82. doi: 10.1016/j.autrev.2006.10.004 17289554

[71] Al-Maleki AR Mariappan V Vellasamy KM Tay ST Vadivelu J. Altered Proteome of Burkholderia Pseudomallei Colony Variants Induced by Exposure to Human Lung Epithelial Cells. PloS One (2015) 10(5):e0127398. doi: 10.1371/journal.pone.0127398 25996927 PMC4440636

[72] Sun P Guo S Sun J Tan L Lu C Ma Z. Advances in In-Silico B-Cell Epitope Prediction. Curr Top Med Chem (2019) 19(2):105–15. doi: 10.2174/1568026619666181130111827 30499399

[73] Larsen JEP Lund O Nielsen M. Improved Method for Predicting Linear B-Cell Epitopes. Immunome Res (2006) 2:2. doi: 10.1186/1745-7580-2-2 16635264 PMC1479323

[74] Ponomarenko J Bui H-H Li W Fusseder N Bourne PE Sette A. ElliPro: A New Structure-Based Tool for the Prediction of Antibody Epitopes. BMC Bioinf (2008) 9:514. doi: 10.1186/1471-2105-9-514 PMC2607291 19055730

[75] Kringelum JV Lundegaard C Lund O Nielsen M. Reliable B Cell Epitope Predictions: Impacts of Method Development and Improved Benchmarking. PloS Comput Biol (2012) 8(12):e1002829. doi: 10.1371/journal.pcbi.1002829 23300419 PMC3531324

[76] Singh H Ansari HR Raghava GPS. Improved Method for Linear B-Cell Epitope Prediction Using Antigen’s Primary Sequence. PloS One (2013) 8(5):e62216. doi: 10.1371/journal.pone.0062216 23667458 PMC3646881

[77] Safavi A Kefayat A Mahdevar E Abiri A Ghahremani F. Exploring the Out of Sight Antigens of SARS-CoV-2 to Design a Candidate Multi-Epitope Vaccine by Utilizing Immunoinformatics Approaches. Vaccine (2020) 38(48):7612–28. doi: 10.1016/j.vaccine.2020.10.016 PMC7546226 33082015

[78] Devi YD Devi A Gogoi H Dehingia B Doley R Buragohain AK. Exploring Rotavirus Proteome to Identify Potential B- and T-Cell Epitope Using Computational Immunoinformatics. Heliyon (2020) 6(12):e05760. doi: 10.1016/j.heliyon.2020.e05760 33426322 PMC7779714

[79] Liu T Shi K Li W. Deep Learning Methods Improve Linear B-Cell Epitope Prediction. BioData Min (2020) 13:1. doi: 10.1186/s13040-020-00211-0 32699555 PMC7371472

[80] B Cell Help. Available at: http://tools.iedb.org/bcell/help/#Bepipred-2.0.

[81] Eickhoff CS Terry FE Peng L Meza KA Sakala IG Van Aartsen D. Highly Conserved Influenza T Cell Epitopes Induce Broadly Protective Immunity. Vaccine (2019) 37(36):5371–81. doi: 10.1016/j.vaccine.2019.07.033 PMC6690779 31331771

[82] Grassl N Kulak NA Pichler G Geyer PE Jung J Schubert S. Ultra-Deep and Quantitative Saliva Proteome Reveals Dynamics of the Oral Microbiome. Genome Med (2016) 8:44. doi: 10.1186/s13073-016-0293-0 27102203 PMC4841045

[83] Zhang X Chen W Ning Z Mayne J Mack D Stintzi A. Deep Metaproteomics Approach for the Study of Human Microbiomes. Anal Chem (2017) 89(17):9407–15. doi: 10.1021/acs.analchem.7b02224 28749657

[84] Blakeley-Ruiz JA Erickson AR Cantarel BL Xiong W Adams R Jansson JK. Metaproteomics Reveals Persistent and Phylum-Redundant Metabolic Functional Stability in Adult Human Gut Microbiomes of Crohn’s Remission Patients Despite Temporal Variations in Microbial Taxa, Genomes, and Proteomes. Microbiome (2019) 7:18. doi: 10.1186/s40168-019-0631-8 30744677 PMC6371617

[85] Long S Yang Y Shen C Wang Y Deng A Qin Q. Metaproteomics Characterizes Human Gut Microbiome Function in Colorectal Cancer. NPJ Biofilms Microbiomes (2020) 6:14. doi: 10.1038/s41522-020-0123-4 32210237 PMC7093434

[86] Park SK (Robin) Jung T Thuy-Boun PS Wang AY Yates JR Wolan DW. ComPIL 2.0: An Updated Comprehensive Metaproteomics Database. J Proteome Res (2019) 18(2):616–22. doi: 10.1021/acs.jproteome.8b00722 PMC7767584 30525664

[87] Perez-Riverol Y Csordas A Bai J Bernal-Llinares M Hewapathirana S Kundu DJ. The PRIDE Database and Related Tools and Resources in 2019: Improving Support for Quantification Data. Nucleic Acids Res (2019) 47(Database issue):D442–50. doi: 10.1093/nar/gky1106 PMC6323896 30395289

